# Effect of opioid-free anaesthesia on post-operative period in cardiac surgery: a retrospective matched case-control study

**DOI:** 10.1186/s12871-019-0802-y

**Published:** 2019-07-31

**Authors:** Pierre-Grégoire Guinot, Alexandra Spitz, Vivien Berthoud, Omar Ellouze, Anis Missaoui, Tiberiu Constandache, Sandrine Grosjean, Mohamed Radhouani, Jean-Baptiste Anciaux, Jean-Philippe Parthiot, Jean-Pierre Merle, Nicolas Nowobilski, Maxime Nguyen, Belaid Bouhemad

**Affiliations:** Department of Anaesthesiology and Critical Care Medicine, Dijon University Medical Centre, 21000 Dijon, France

**Keywords:** Anesthetics, Lidocaine, Intravenous, Propofol, Opioid, Cardiac surgery, Outcomes

## Abstract

**Background:**

No study has been conducted to demonstrate the feasibility of an opioid-free anesthesia (OFA) protocol in cardiac surgery to improve patient care. The aim of the present study was to evaluate the effect of OFA on post-operative morphine consumption and the post-operative course.

**Methods:**

After retrospectively registering to clinicaltrial.gov (NCT03816592), we performed a retrospective matched cohort study (1:1) on cardiac surgery patients with cardiopulmonary bypass between 2018 and 2019. Patients were divided into two groups: OFA (lidocaine, dexamethasone and ketamine) or opioid anaesthesia (OA) (sufentanil). The main outcome was the total postoperative morphine consumption in the 48 h after surgery. Secondary outcomes were rescue analgesic use, a major adverse event composite endpoint, and ICU and hospital length of stay (LOS).

**Results:**

One hundred ten patients were matched (OFA: *n* = 55; OA: n = 55). On inclusion, demographic and surgical data for the OFA and OA groups were comparable. The total morphine consumption was higher in the OA group than in the OFA group (15 (6–34) vs 5 mg (2–18), *p* = 0.001). The pain score during the first 48 post-operative hours did not differ between the two groups. Creatinine values did not differ on the first post-operative day (80 (IQR: 66–115) vs 77 mmol/l (IQR: 69–95), *p* = 0.284). Incidence of the composite endpoint was lower in the OFA group (25 patients (43%) vs 38 patients (68%), *p* = 0.021). The time to extubation and the ICU stays were shorter in the OFA group (3 (1–5) vs 5 (3–6) hours, p = 0.001 and 2 (1–3) vs 3 (2–5) days, *p* = 0.037).

**Conclusion:**

The use of OFA was associated with lower morphine consumption. OFA might be associated with shorter intubation time and ICU stays. Further randomized studies are needed to confirm these results.

**Trial registration:**

This study was retrospectively registered to ct2 (identifier: NCT03816592) on January 25, 2019.

## Background

Since the 1960s, the systematic administration of opioids has been considered one of the pillars of modern anaesthesia [1]. The use of opioid analgesics has become widespread with the development of new opioid agents. Their use is based on their antinociceptive effects, the control of the autonomic nervous system (ANS) responses to surgical stress, and their induced hypnotic reduction [2]. However, the principle underlying the administration of opioids during anaesthesia has only recently been called into question. Opioids have a number of adverse effects that limit their effectiveness in perioperative care, the most relevant being respiratory depression, gastrointestinal alterations, hyperalgesia, inflammation modulation, and immunologic modulation [3]. Moreover, the recent opioid epidemic, due in part to persistent use of perioperative opioids, raises questions about the systematic administration of opioids during anaesthesia and the development of new non-opioid strategies.

Opioid-free anaesthesia (OFA) is a long-standing concept. It is based on the fact that a sympathetic reaction evidenced by hemodynamic changes in an anesthetised patient does not systematically reflect pain. In addition, a sleeping patient will not recall pain, while hormonal stress and sympathetic and inflammatory reactions can be controlled by therapeutic classes other than opioids [4, 5]. There is an increasing body of literature on OFA, demonstrating its feasibility with a decrease of post-operative morphine consumption and improvement of postoperative well being [6–9]. Several OFA protocols have also been published [10]. The most commonly used nonopioid agents are lidocaine, dexamethasone, and ketamine [10], and all have been studied separately in cardiac surgery. Murphy et al. demonstrated that the administration of dexamethasone decreased morphine consumption and the ICU length of stay [11]. Ketamine was shown to have analgesic effects and opioid-sparing effects [12]. Lidocaine has demonstrated analgesic and opioid-sparing effects in cardiac and non-cardiac surgery [13, 14], and additional studies have found cardioprotective and/or neuroprotective effects [15, 16]. More specifically, the use of lidocaine has been associated with a decrease in arrhythmias and a non-constant improvement in postoperative cognitive functions. All these studies were performed with opioid anaesthesia (OA). In non-cardiac surgery, OFA was demonstrated to be associated with lower post-operative opioid use, and better respiratory outcomes [6, 7, 17]. To our knowledge, no study has evaluated the effect of OFA on morphine consumption and the post-operative course in cardiac surgery patients.

The main objective of the present study was to demonstrate that, compared with OA, OFA lowers postoperative morphine consumption. In addition, we evaluated the effect of OFA on operative hemodynamic stability, postoperative complications assessed by a composite criterion and the length of stay (LOS) in the ICU and in hospital.

## Methods

### Patients

We performed a retrospective, open-label, matched (1:1), single-centre study in a tertiary university hospital (Dijon, France) between 2018 and 2019. All patients were included within this time frame. The study was recorded on January 25, 2019 to clinicaltrial.gov (NCT03816592). Our team started using an OFA strategy for cardiac surgery in 2017. Consequently, the number of patients receiving OFA has increased steadily since 2017. In practical terms, OFA administration was left to the discretion of the attending anesthetist regardless of a patient’s co-morbidities and surgical risk. The study was performed in accordance with the ethical standards outlined in the 1964 Declaration of Helsinki. As the study was observational and used existing, routinely collected data, and in compliance with French law (loi Jardé n° 2012–300) informed consent was not required. We submitted the protocol study to the “Délégation à la Recherche et à l’Innovation (DRCI) », CHU de Dijon, Dijon, France, and followed the MR004 (méthodologie de référence 004) in accordance with the national commission for data protection (CNIL) guidelines. The present report was drafted in line with the STROBE statement [18].

The main inclusion criteria were as follows: age 18 or over, cardiac surgery with the use of cardiopulmonary bypass (CPB) (coronary artery bypass grafting (CABG), the surgical correction of valve disease (aortic, mitral), combined surgery (CABG and valve disease), ascending aortic disease, and left ventricular assist device implantation). The exclusion criteria were: off-pump cardiac surgery, preoperative analgesic use, gabapentin use, antidepressant therapy and preoperative cognitive dysfunction.

### Operative management

Maintenance or withdrawal of preoperative medications adhered to international guidelines. Anaesthesia and cardiopulmonary bypass procedures were standardised for all patients. In the OA group, anaesthesia was induced with an intravenous bolus of ketamine (0.3–0.5 mg/kg), propofol (0.4–2 mg/kg) and sufentanil (0.5 ng/ml) until the loss-of-eyelash reflex. Sufentanil was continuously administered using Schneider’s target-controlled infusion model. All OA patients had loco-regional analgesia by serratus anterior plane block (thoracotomy, levobupivacaine 0.125 mg/ml, 0.5 ml/kg) or a continuous parasternal infusion of a local anaesthetic (sternotomy, levobupivacaine 0.125 mg/ml continuous infusion 8 ml/h for 48 h). None of the OA patients received intravenous lidocaine. In the OFA group, anaesthesia was induced with an intravenous bolus of dexamethasone (0.1 mg kg^− 1^), intravenous bolus of ketamine (0.3–0.5 mg kg^− 1^), intravenous bolus of lidocaine (1.5 mg kg^− 1^ bolus 15 min before the start of propofol) and propofol (0.4–2 mg kg^− 1^) until the loss-of-eyelash reflex. Lidocaine was continuously administered at 1.5 mg/kg/h until the end of surgery.

In both groups, tracheal intubation was facilitated with cisatracurium (0.15 mg kg^− 1^), and neuromuscular blockade was ensured using intermittent bolus of cisatracurium to reach adequate muscle relaxation measured by peripheral nerve stimulation. Sufentanil and lidocaine were discontinued at the end of surgery. In both groups, anaesthesia was maintained by target-controlled infusions of propofol (started at 2–4 ng ml^− 1^). Sedation was titrated using the bispectral index (Covidien, Boulder, CO, USA) to obtain a value between 40 and 60. Arterial hypertension (systolic arterial pressure > 140 mmHg) was treated with esmolol in case of tachycardia (heart rate > 80 bpm) or urapidil/nicardipine if the heart rate dropped below 80 bpm.

Cardiopulmonary bypass was conducted with a heart-lung machine (Stockert Sorin S5 Heart Lung, Milan, Italy) at a target blood flow of 2.4 l min^− 1^ m^− 2^. The mean arterial blood pressure (MAP) was maintained at more than 65 mmHg by increasing the pump flow rate or, if required, by administering a bolus of phenylephrine (100 μg) or norepinephrine (5 μg). The CPB circuit was primed with 1500 ml of crystalloids (Plasma-Lyte®; Baxter, Lessines, Belgium) and 5000 ui of heparin. After systemic heparinization (300 ui kg^− 1^) to obtain a hemochron level of 400 s, median sternotomy or thoracotomy was performed and aortic and right auricular cannulations were started. Normoglycemia was maintained using intravenous insulin (intravenous bolus of 5–10 ui) if necessary. Patients with a haemoglobin value below 8 g dl^− 1^ received homologous red blood cell transfusions. Heparin was reversed with protamine at a 1:1 ratio.

### ICU management

At the end of surgery, all patients were sedated and the lungs were mechanically ventilated until haemodynamic stability and normothermia were obtained and blood loss was considered acceptable (less than 1 ml kg^− 1^ h^− 1^). Tracheal extubation was done according to the French guidelines [19]. Patients were managed by a team of physicians trained in postoperative cardiac surgical care which included a cardiologist.

Analgesia was standardised and comprised of intravenous paracetamol (1 g every 6 h) and patient-controlled morphine analgesia. Before extubation, all patients received 1 g of acetaminophen. Immediately after, all patients received titrated intravenous morphine with a bolus of 2 to 3 mg until a score of 3 or less was obtained on the visual analogue pain scale. Patient-controlled analgesia morphine was then started as follows: 1 mg bolus, refractory period of 7 min, maximum dose of 20 mg every 4 h without continuous infusion. The use of complementary analgesics was left to the discretion of the attending physician. According to our institutional protocol, complementary analgesia comprised the use of ketoprofen (50 mg), tramadol (50 mg), and nefopam (20 mg). Pain was assessed every 4 h during the ICU stay with the visual analogue scale. Non-invasive ventilation was left to the discretion of attending physician. Common indications were high-risk patients, atelectasis, hypoxemia, hypercapnia and acute respiratory failure. Patients were discharged from ICU at the discretion of their attending physician. The patients’ electrocardiogram, pulse oxygen saturation and central venous blood pressure were continuously monitored. The scheduled blood tests included arterial/venous blood gas measurements on admission to the ICU, and then several times a day on request by the attending physician.

The following variables were continuously recorded in the institutional database: age, gender, body weight, height, personal medical history, ASA score, EuroSCORE2, Euroscore, type of cardiac surgery, the preoperative left ventricular ejection fraction, the duration of CPB, the duration of aortic clamping, the need for intraoperative blood transfusion, norepinephrine, dobutamine, the use of a antihypertensive agent (nicardipine, urapidil), the use of a short acting beta-blocker (esmolol), troponin values, creatinine value, time to extubation (hours), any occurrence of complications during the stay in the ICU or in the hospital, and the LOS in the ICU.

The primary endpoint was the cumulative dose of postoperative morphine in the first 48 h (in milligrams). The secondary endpoints were: analgesic rescue, a composite endpoint of major adverse events (new onset of atrial fibrillation or flutter, second or third atrio-ventricular blockade, stroke, acute kidney injury, confusion, reintubation, non-invasive ventilation support, and in-hospital death), fluid expansion (ml), total propofol dose (mg), antihypertensive agent use, vasoplegia syndrome, catecholamine use, troponin Ic (ng ml^− 1^), creatinine (mmol l^− 1^), ICU LOS (days), and hospital LOS (days). The secondary composite endpoint was assessed during the hospital stay. All data was extracted from our institutional database and collected by a physician who was not involved in the care of the study patients.

### Statistical analysis

The trial was designed to investigate the potential superiority of OFA in terms of postoperative morphine consumption. According to the studies of Berthoud et al., we calculated that 55 patients per group would be sufficient to demonstrate a 10 mg difference in morphine consumption (with a mean consumption of 18 mg) with a power of 0.8 and an alpha risk of 0.05 [20, 21]. Based on known factors associated with postoperative pain and operative course, the database was matched (1:1) on age, body mass index, Euroscore 2, and type of surgery (sternotomy/thoracotomy) [22, 23]. Normal distribution was assessed using the Shapiro-Wilk test. Data are expressed as medians (interquartile range). The Wilcoxon test was used for the comparison of matched continuous variables, and the Cochran-Mantel-Haenszel test with odds ratios (OR) was used or the comparison of categorical variables [21]. The threshold for statistical significance was set at *p* < 0.05. Statistical analyses were performed with SPSS 24 (IBM, France).

## Results

**Fig. 1:**
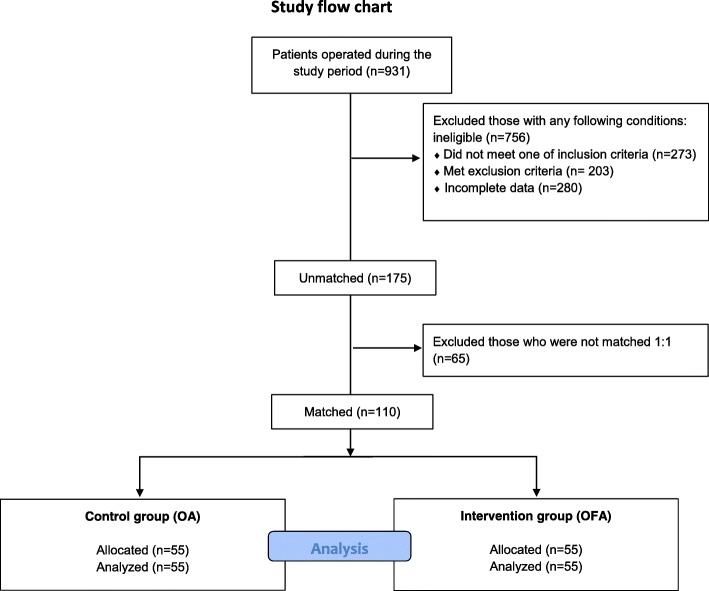
Flow chart diagram

Of the 931 patients operated during the study period, 110 were matched and analysed (Fig. 1). The intervention and control groups did not differ significantly in their demographic characteristics and cardiac surgery type (Tables 1 and 2). In the overall study population, the mean age was 69 years (IQR: 63–74, males: *n* = 78), the median EuroSCORE2 was 1.6 (IQR: 0.89–3.01) and the median EuroScore was 6 (IQR: 4.9–8.3).

**Table 1:** Demographic characteristics of the population. Data are expressed as median (interquartile range), or number (%). The *p*-value always refers to comparison between the OFA group and the OA group

Variables	OFA group (*n* = 55)	OA group (n = 55)	*P*-value
Age, (years)	69 (62–74)	69 (65–74)	0.066
Gender (M), n (%)	43 (78)	35 (64)	0.115
Body mass index (kg m ^−2^)	26 (24–30)	26 (23–30)	0.162
ASA, n (%)			
2	3 (5)	7 (12)	
3	41 (75)	38 (69)	
4	11 (20)	10 (19)	
EuroSCORE II (%)	1.51 (0.98–3.1)	1.69 (0.87–3.01)	0.805
Euroscore (%)	6 (5–9)	6 (3–8)	0.238
Medical history, n (%)			
Coronary disease	31 (56)	27 (49)	0.571
Arrhythmia	8 (14)	13 (24)	0.332
Dyslipidaemia	31 (56)	34 (62)	0.556
Smoking history	23 (42)	19 (35)	0.556
High blood pressure	42 (76)	43 (77)	1
Diabetes	15 (27)	15 (27)	1
Chronic renal insufficiency	12 (22)	7 (13)	0.276
Stroke	4 (7)	4 (7)	1
Obstructive sleep apnoea	7 (13)	7 (13)	1
Treatment, n (%)			
Beta blocker	34 (62)	34 (62)	1.00
Calcic channel blocker	9 (16)	6 (11)	0.581
Aspirin	32 (58)	28 (49)	0.596
Clopidogrel	2 (4)	6 (11)	0.261
Angiotensin enzyme converting inhibitor	21 (38)	23 (42)	0.832
Statin	32 (58)	34 (62)	0.832
Diuretics	21 (38)	19 (35)	0.850
Left Ventricular Ejection Fraction (%)	60 (51–65)	60 (50–65)	0.278
Creatinine (mmol l^−1^)	83 (72–96)	84 (70–98)	0.558

**Table 2:** Operative characteristics of the population

Variables	OFA group (n = 55)	OA group (n = 55)	OR (CI_95%_)	*P*-value
Type of surgery				
Valvular surgery	20 (34)	22 (38)		0.806
Coronary artery bypass graft	11 (22)	9 (19)		0.845
Combined	21 (38)	21 (38)		1
Ascending aorta	1 (2)	1 (2)		1
LVAD	2 (4)	2 (4)		1
Sternotomy/thoracotomy	40/15	39/16		1
Urgent, n (%)	13 (23)	10 (18)		0.641
Anaesthesia time (min)	303 (268–360)	308 (288–360)		0.409
Cardiopulmonary bypass time (min)	118 (102–148)	119 (101–149)		0.505
Aortic cross clamp time (min)	84 (66–119)	94 (72–114)		0.383
Propofol				
mg	1351 (922–1840)	1145 (740–1634)		**0.008**
mg kg^−1^	17.5 (13.4–22.6)	15.5 (10.9–18.5)		**0.045**
Sufentanil				
Gamma	0	89 (75–120)		**0.0001**
Gamma kg^−1^	0	1.12 (0.93–1.56)		0.062
Anti hypertensive agents, n (%)				
Urapidil	28 (50)	12 (22)	3.716 (1.620–8.522)	**0.003**
Median dose (mg)	15 (10–20)	10 (5–15)		0.713`
Nicardipin	6 (11)	10 (18)	0.551 (0.185–1.639)	0.419
Median dose (mg)	1.5 (1–2)	1 (0.5–2.5)		0.157
Vasopressor, n (%)				
Ephedrine	28 (51)	40 (73)	0.389 (0.176–0.861)	**0.032**
Median dose (mg)	15 (9–28)	26 (15–30)		0.130
Phenylephrine	36 (67)	31 (56)	1.467 (0.679–3.168)	0.588
Median dose (mg)	0.275 (0.15–0.5)	0.35 (0.2–0.55)		0.272
Norepinephrine	40 (73)	30 (55)	2.222 (1.002–4.927)	0.076
Median cumulated dose (mg)	1.24 (0.35–2.4)	1.34 (0.41–2.46)		0.092
Esmolol, n (%)	11 (20)	1 (2)	13.5 (1.677–108.652)	**0.006**
Median dose (mg)	30 (10–46)	73 (73–73)		
Dobutamine, n (%)	7 (13)	8 (15)	0.857 (0.288–2.551)	1
Total fluid administration				
Cumulated in ml	1250 (1000–1500)	1500 (1000–2000)		0.060
Cumulated in ml kg^−1^	15.2 (10.7–21.4)	21.4 (12.5–27.7)		0.015
Transfusion, n (%)	13 (24)	13 (24)	1 (0.439–2.277)	1

Data are expressed as median (interquartile range), or number (%). The p-value always refers to comparison between the OFA group and the OA group

### Analgesia evaluation

The total postoperative morphine dose was significantly different in the OFA and OA groups: 5 (IQR: 2 to 18) vs 15 mg (IQR: 6–34), *p* = 0.001 (Table 3). However, complementary analgesia did not differ significantly: ketoprofen (18% vs 16%, OR = 1.136, *p* = 1 and 150 (IQR: 50–262) vs 125 mg (IQR: 50–263), p = 1)), nefopam (20% vs 13%, OR = 1.714, *p* = 0.441 and 40 (IQR: 20–100) vs 20 mg (20–33), *p* = 0.317), and tramadol (31% vs 31%, OR = 1, p = 1, and 100 (IQR: 50–150) vs 100 mg (IQR: 50–150), p = 1)) were used in equal measures. The pain scores were also similar in the two groups (Table 3).

**Table 3:** Post-operative course of primary (total postoperative morphine consumption) and secondary endpoints (complementary analgesia, postoperative complications, ICU and hospital stays)

Variables	OFA group (n = 55)	OA group (n = 55)	OR (CI_95%_)	*P*-value
Total morphine consumption (mg)	5 (2–18)	15 (6–34)		**0.001**
Additional analgesic (n, %)				
Tramadol	17 (31)	17 (31)	1 (0.445–2.245)	1
Total median dose (mg)	150 (50–262)	125 (50–263)		1
Ketoprofen	10 (18)	9 (16)	1.136 (0.422-3.056)	1
Total median dose (mg)	100 (50–150)	100 (50–150)		1
Nefopam	11 (20)	7 (13)	1.714 (0.611–4.812)	0.441
Total median dose (mg)	40 (20–100)	20 (20–33)		0.317
Visual analog score				
Extubation	0 (0–2)	0 (0–3)		0.324
12 h after ICU admission	0 (0–0)	0 (0–1)		0.777
First postoperative day	0 (0–2)	0 (0–2)		0.665
Second postoperative day	0 (0–2)	0 (0–2)		0.874
Vomiting (n, %)	5 (9)	9 (16)	0.511 (0.160–1.637)	0.393
Creatinine (mmol l^−1^)				
at admission to ICU	83 (72–106)	76 (64–91)		0.105
on first postoperative day	80 (66–115)	77 (69–95)		0.284
Troponin Ic (ng ml^−1^)				
at admission to ICU	6.2 (3.7–10)	7.5 (2.9–20)		0.091
24 h after surgery	4.7 (2.6–8.2)	7 (3.1–16)		0.091
PaO_2_				
at admission to ICU	162 (113–195)	155 (114–181)		0.479
24 h after surgery	99 (83–122)	115 (89–138)		0.084
FiO_2_				
at admission to ICU	50 (30–60)	50 (50–60)		**0.005**
24 h after surgery	28 (25–32)	32 (30–36)		**0.018**
PaO_2_/FiO_2_ ratio				
at admission to ICU	357 (275–458)	290 (213-372)		**0.006**
24 h after surgery	355 (261–420)	322 (265–435)		0.919
Time to extubation (hours)	3 (1–5)	5 (3–6)		**0.001**
Catecholamine use, n (%)				
Norepinephrine	26 (32)	31 (38)	0.694 (0.328–1.471)	0.447
Dobutamine	9 (16)	9 (16)	0.766 (0.277–2.115)	0.798
Transfusion, n (%)				
Red blood cell	16 (29)	18 (33)	0.843 (0.375–1.895)	0.837
Endpoint composite score, n (%)	**25 (43)**	**38 (66)**	**0.373 (0.171–0.813)**	**0.021**
Confusion	4 (7)	10 (19)	0.353 (0.103–1.204)	0.151
Stroke	1 (2)	0		1
Seizure	1 (2)	0		1
Atrial fibrillation,	15 (27)	22 (40)	0.563 (0.252–1.254)	0.228
Ventricular tachycardia or fibrillation	1 (2)	3 (5)	0.315 (0.032–3.125)	0.599
Atrio-ventricular block requiring pacemaker implantation	2 (4)	1 (2)	2.038 (0.179–23.151)	1
Acute renal failure	12 (22)	14 (26)	0.817 (0.338–1.974)	0.823
Reintubation	1 (2)	3 (6)	0.321 (0.032–3.186)	0.612
Non-invasive ventilation	15 (27)	27 (49)	0.389 (0.176–0.861)	**0.032**
Mortality	1 (2)	1 (2)	1 (0.061—16.401)	1
ICU stays (days)	2 (1–3)	3 (2–5)		**0.037**
Hospital stays (days)	8 (7–15)	10 (8–14)		0.790

Data are expressed as median (25th to 75th percentiles), or number (%). The p-value always refers to comparison between the OFA group and the OA group

### Secondary endpoints

Operative data differed between groups in terms of vasopressor use, antihypertensive drug use, and total fluid infused (Table 2). More patients in the OFA group were treated with urapidil (50% vs 22%, *p* = 0.003) whereas more patients in the OA group were treated with ephedrine (73% vs 52%, *p* = 0.032). The time to extubation was shorter in the OFA group (3 (IQR: 1–5) vs 5 h (IQR: 3–6), p = 0.001) (Table 3). The composite endpoint was less common in the OFA group (25 (43%) vs 38 patients (68%), OR = 0.373, *p* = 0.021) along with a lower use of non-invasive ventilation (27% vs 49%, OR = 0.389, p = 0.032). Creatinine values did not differ on the first post-operative day (80 (IQR: 66–115) vs 77 mmol/l (IQR: 69–95), *p* = 0.284). The incidence of cardiac and renal outcomes were similar. The OFA group spent less time in the ICU (2 (IQR: 1–3) vs 3 days (IQR: 2–5), *p* = 0.037) though the length of the hospital stay did not differ (8 (IQR: 7–15) vs 10 days (IQR: 8–14), *p* = 0.790).

## Discussion

In the present study, we found that OFA was associated with: (1) lower postoperative morphine consumption; (2) higher operative use of antihypertensive agents; (3) a decrease of orotracheal intubation time and the use of non-invasive respiratory support; and (4) shorter ICU stays.

To date, two published case reports and one retrospective study have reported on the use of OFA study in thoracic surgery [7, 24, 25], but no study has focused specifically on the use of OFA in cardiac surgery. In non-cardiac surgery with OFA, previous studies have demonstrated decreased postoperative pain scores and opioid consumption [6, 8, 9]. In cardiac surgery, the studies evaluating loco-regional analgesia have demonstrated a decrease in morphine consumption [20]. Despite its retrospective design, the present study confirmed the feasibility of OFA in cardiac surgery. Because OFA is based on the opioid avoidance with a multimodal analgesic treatment, it is associated with lower postoperative morphine consumption and fewer of the adverse effects that result from opioid use. The postoperative pain score did not differ between groups, indicating that OA and OFA provided comparable analgesia.

Yet one important question remains: what is the analgesic/anaesthetic effect of OFA during surgery [26]? OFA is an anaesthesia strategy that replaces opioids (balanced general anesthesia) with non-opioid drugs (multimodal general anesthesia). Opioids are usually administered during anesthesia for their antinociceptive effects, to control the responses of the autonomic nervous system (ANS) to surgical stress, and for their hypnotic effect. Our approach to OFA was based on published literature which demonstrates that each of the effects associated with opioids can be obtained with lidocaine (analgesic, hypnotic, and ANS control), dexamethasone (analgesic) and ketamine (analgesic and hypnotic) [11–13]. The lidocaine dosing regimen was based on the literature published since the 1990s that has demonstrated a safety profile with continuous infusion of lidocaine during cardiac and non-cardiac surgeries [27, 28]. Lidocaine has effects that depend on the total dose, and detrimental effects can be seen with elevated doses of lidocaine [27]. But lidocaine also has beneficial effects during surgery, providing (a) an anti-inflammatory effect; (b) an increase in the cardioprotective effect of cardioplegia; (c) a decrease in the risk of arrhythmias; and (d) a decrease in the risk of brain inflammation [15, 16, 28, 29].

OFA may be associated with certain adverse effects such as a higher incidence of blood pressure events or adverse effects resulting from toxic plasma levels. We observed a trend towards higher use of norepinephrine and anti-hypertensive agents that may be the result of several factors: the increasing use of propofol, the half-life of urapidil/nicardipine (mostly used before CPB), and the vasoactive effect of lidocaine. Several studies have already underlined the growing incidence of hypertensive episodes and the higher use of anti-hypertensive agents [6, 7]. Bakan et al. also found that a higher total dose of propofol was needed to maintain OFA [6]. According to the literature, the dosage regimen used in the present study may be associated with low to moderate plasma values of lidocaine, which are associated with vasoconstriction [27]. Because blood pressure may be high, physicians frequently use anti-hypertensive agents. On the contrary, because the hypnotic effect of lidocaine may be less marked than that of opioids, physicians increase propofol doses which can potentially cause arterial hypotension. Finally, the combination of high-dose propofol and the increasing use of anti-hypertensive agents may increase the need for vasopressors during CPB. Accordingly, we did not demonstrate a higher incidence of postoperative vasoplegic syndrome. To date, we do not have observed clinical signs of local anaesthetic toxicity (arrhythmia, atrial-ventricular block, seizure) with our protocol, which confirms the existing data on lidocaine plasma levels [27–29].

Though we did not use dexmedetomidine in our protocol because it is not available in our department, the uses of an alpha_2_ agent may have several advantages. First, the combination of dexmedetomidine and lidocaine was shown to provide better postoperative pain relief than the use of each agent individually [14]. Also, in cardiac surgery, a recent meta-analysis confirmed that dexmedetomidine provided good hemodynamic stability during surgery with less tachycardia and arterial hypertension [30]. Moreover, studies suggest a positive effect on confusion and atrial fibrillation, with a shorter time to extubation and a shorter ICU length of stay [31, 32]. According to the literature, the combination of lidocaine and dexmedetomidine should improve hemodynamic stability and decrease the need for antihypertensive agents.

We demonstrated that OFA resulted in lower intubation time and use of non-invasive ventilation. The respiratory effects may be explained by the avoidance of opioids and better pain relief. In our experience, patients anesthetized with OFA have a shorter period of respiratory inhibition during surgery than patients anesthetized with opioids. Spontaneous breathing returns sooner following OFA and patients seem to become alert more quickly after orotracheal extubation. In our results, oxygen requirements and non-invasive ventilation was lower in the OFA group. These effects may be associated with the respiratory depression and cognitive dysfunction that are well-known effects of opioid sedation [8, 33].

The present study had several limitations. Our study was a single retrospective study, which implies a certain number of design-related limitations. Despite protocol management for sedation and analgesia, bias may have been introduced by the attending physician and nursing staff. In addition, some patients received analgesia in addition to patient-controlled morphine analgesia, suggesting postoperative multimodal analgesia. Nevertheless, the protocol for postoperative intravenous analgesic use was similar in the two groups. Similar limitations should be mentioned for non-invasive ventilation and ICU stays, which were left to the discretion of the attending physician. Only controlled prospective randomized studies can confirm the present results. Moreover, further studies are needed to determine the optimal associations, dosages, and infusion protocols during cardiac surgery. We have included two types of surgery (sternotomy and thoracotomy) than could be not equivalent in term of pain. Because the matching was based on this aspect, the two groups did not differ in term of type of surgery. The relatively small number of patients in our population might also limit our study’s external validity. Nevertheless, we calculated a sample size based on morphine consumption, which has been demonstrated to be associated with OFA, and we included a mixed cardiac surgery population. We believe our results demonstrate the feasibility of OFA in several cardiac surgery subtypes. Our team currently uses OFA in cardiac surgery on a daily basis without any restrictions other than contraindications to lidocaine use.

## Conclusion

The present study demonstrated that OFA was associated with a decrease in postoperative morphine consumption. OFA might have beneficial effects of on the post-operative course of patients undergoing cardiac surgery with CBP. Further randomized studies are needed to confirm these results.

## Data Availability

All data and related metadata underlying the findings reported in our study are provided as part of the submitted article. Additional data is available on reasonable request from the corresponding author.

## Abbreviations

CPB: Cardiopulmonary bypass
ICU: Intensive care unit
LOS: Length of stay
MAP: Mean arterial pressure
OA: Opioid anesthesia
OFA: Opioid-free anesthesia
